# Simultaneous initiation of finerenone and empagliflozin across the spectrum of kidney risk in the CONFIDENCE trial

**DOI:** 10.1093/ndt/gfaf160

**Published:** 2025-08-31

**Authors:** Muthiah Vaduganathan, Jennifer B Green, Hiddo J L Heerspink, Sung Gyun Kim, Johannes F E Mann, Janet B McGill, Amy Mottl, Masaomi Nangaku, Julio Rosenstock, Peter Rossing, Li Li, Na Li, Katja Rohwedder, Charlie Scott, Rajiv Agarwal

**Affiliations:** Division of Cardiovascular Medicine, Brigham and Women's Hospital and Harvard Medical School, Boston, MA, USA; Division of Endocrinology, Department of Medicine and Duke Clinical Research Institute, Duke University School of Medicine, Durham, NC, USA; Department Clinical Pharmacy and Pharmacology, University of Groningen and University Medical Center Groningen, Groningen, The Netherlands; Division of Nephrology, Hallym University Sacred Heart Hospital, Anyang, South Korea; KfH (Kuratorium für Dialyse und Nierentransplantation) Kidney Center, Munich, Germany; Department of Nephrology and Hypertension, Friedrich Alexander University, Erlangen, Germany; Division of Endocrinology, Metabolism and Lipid Research, Washington University in St Louis, St Louis, MO, USA; University of North Carolina (UNC) Kidney Center, UNC School of Medicine, Chapel Hill, NC, USA; Division of Nephrology and Endocrinology, The University of Tokyo Graduate School of Medicine, Tokyo, Japan; Velocity Clinical Research at Medical City, Dallas, TX, USA; Steno Diabetes Center Copenhagen and University of Copenhagen, Copenhagen, Denmark; Bayer AG, Berlin, Germany; Bayer Healthcare, Beijing, China; Bayer AG, Berlin, Germany; Bayer Healthcare Inc., Whippany, NJ, USA; Division of Nephrology, Richard L. Roudebush VA Medical Center, Indianapolis Indiana University School of Medicine, Indianapolis, USA

**Keywords:** clinical trials, mineralocorticoid receptor antagonists, sodium-glucose co-transporter-2 inhibitors

## Abstract

**Background:**

The CONFIDENCE (COmbinatioN effect of FInerenone anD EmpaglifloziN in participants with CKD and type 2 diabetes using a UACR Endpoint) trial investigated the safety and efficacy of simultaneously initiating finerenone and empagliflozin for patients with chronic kidney disease (CKD) and type 2 diabetes. This prespecified analysis aimed to determine if the predicted risk of kidney disease progression, based on KDIGO risk categories, influenced the benefits and safety of this combination therapy.

**Methods:**

The double-blind, double-dummy trial randomized 818 adults with CKD and type 2 diabetes [urine albumin–creatinine ratio (UACR) ≥100 to <5000 mg/g] to receive once-daily finerenone plus empagliflozin, finerenone alone or empagliflozin alone, all in addition to a renin–angiotensin system inhibitor. The relative change in UACR from baseline to day 180 (primary endpoint) and a >30% reduction in UACR (secondary endpoint) across KDIGO risk categories was assessed.

**Results:**

At baseline, among 781 with available data, 11.3% of participants were classified as low/moderate risk, 29.6% as high risk and 59.2% as very high risk. At 180 days, combination therapy significantly reduced UACR levels across all KDIGO risk categories (low/moderate: –61.7%; high: –60.7%; very high: –52.4%). This reduction was consistently greater than that achieved with either monotherapy alone. More than half of patients on combination therapy experienced UACR reductions of >30% (low/moderate: 58.1%; high: 74.2%; very high: 70.6%), again outperforming monotherapies across all risk groups. While hyperkalemia was more common with combination therapy, early eGFR declines (>30% within 30 days) were less frequent in individuals with higher KDIGO risk compared with lower risk. Overall, the safety profile of combination therapy remained consistent across all KDIGO risk categories, with no unexpected safety signals.

**Conclusions:**

The CONFIDENCE trial demonstrates that the relative efficacy and safety of simultaneous finerenone and empagliflozin combination therapy are consistent across a wide spectrum of predicted kidney disease risk.

**Clinical Trial Registration:**

NCT05254002; EudraCT 2021-003037-11

KEY LEARNING POINTS **What was known:**

- The Kidney Disease: Improving Global Outcomes (KDIGO) classification scheme forecasts risks of kidney disease progression, cardiovascular events, and mortality.
- The KDIGO “heat map” may serve as a shared decision-making tool for clinicians and patients to discuss individualized risk, monitor disease trajectory and response to therapies, and review management options to ameliorate risk.

**This study adds:**

- In the CONFIDENCE trial, the relative efficacy and safety of simultaneous combination therapy with finerenone and empagliflozin were consistent across a broad range of predicted kidney risk.

**Potential impact:**

- Patients with type 2 diabetes and chronic kidney disease across the KDIGO risk spectrum may benefit from simultaneous initiation of combination therapy with the non-steroidal mineralocorticoid receptor antagonist finerenone and a sodium-glucose co-transporter-2 inhibitor.

## INTRODUCTION

Chronic kidney disease (CKD) represents a global health crisis affecting 850 million people [1], with the number of individuals reaching kidney failure projected to rise, especially in low- and middle-income countries [2]. The Kidney Disease: Improving Global Outcomes (KDIGO) classification scheme [3] was developed as a simple, yet comprehensive and effective tool to improve awareness, diagnosis, risk stratification, treatment decision-making and specialist referral for persons with CKD. By juxtaposing two laboratory biomarkers of risk [estimated glomerular filtration rate (eGFR) and urine albumin–creatinine ratio (UACR)], KDIGO categories not only powerfully forecast future risk of kidney failure, but also cardiovascular events, hospitalizations, and mortality [4]. The color gradient offered within the eGFR and UACR grid may also serve as a shared decision-making tool for clinicians and patients to discuss individualized risk, monitor disease trajectory and response to therapies, and review management options to ameliorate risk.

The 2024 KDIGO guidelines suggest that any person with CKD identified with moderate or higher risk should be treated with specific risk lowering therapies [3]. In current clinical practice, therapies are often initiated one-at-a-time with further intensification guided by follow-up measurement of safety parameters and UACR [3]. However, in recent years, several therapies [sodium glucose co-transporter-2 inhibitors (SGLT2i), non-steroidal mineralocorticoid receptor antagonists (nsMRA) and glucagon-like peptide-1 receptor agonists (GLP-1RA)] have independently demonstrated potential to slow kidney disease progression and prevent cardiovascular events, when added to renin–angiotensin system inhibitors [5–7]. In light of the broad heterogeneity of the global CKD population, some have advocated for a ‘risk-based’ implementation approach intended to match risk of disease progression with intensity of therapeutic optimization. More specifically, some patients with a high or very high predicted risk of near-term clinical events and kidney failure may theoretically benefit from initiation of early combination therapy with more than one risk lowering therapy [8]. However, few prospective data are available to evaluate the relative risks and benefits of early combination across the spectrum of kidney risk.

The CONFIDENCE (COmbinatioN effect of FInerenone anD EmpaglifloziN in participants with CKD and type 2 diabetes using a UACR Endpoint) trial demonstrated that the upfront simultaneous initiation of the nsMRA finerenone and the SGLT2i empagliflozin was safe and more effective at lowering UACR by 180 days than either therapy alone among patients with type 2 diabetes and CKD [9]. In this prespecified analysis, we examined whether predicted risk of kidney disease progression (based on the KDIGO risk categories) might modify the efficacy and safety of early combination therapy of finerenone and empagliflozin.

## MATERIALS AND METHODS

### The CONFIDENCE trial

The design [10], baseline characteristics [11], protocol [9] and the main results [9] of the CONFIDENCE trial have been previously published. In brief, the CONFIDENCE trial was a double-blind, double-dummy trial that enrolled adults with type 2 diabetes and CKD with albuminuria. Participants were randomized 1:1:1 to simultaneous initiation of finerenone (10 or 20 mg daily) and empagliflozin (10 mg daily) versus finerenone (10 or 20 mg) plus placebo, or versus empagliflozin (10 mg) plus placebo. Finerenone was started at 20 mg once daily when the eGFR was ≥60 mL/min/1.73 m^2^ and 10 mg once daily when the eGFR was <60 mL/min/1.73 m^2^ (with uptitration to 20 mg once daily after 30 days if serum potassium was ≤4.8 mmol/L and if eGFR had not declined by 30% or greater). Randomization was stratified according to the screening eGFR (<60 and ≥60 mL/min/1.73 m²) and UACR (≤850 and >850 mg/g).

In Part A of the trial, qualifying eGFR was required to be 40–90 mL/min/1.73 m², which was subsequently broadened in Part B of the trial (after an independent Data Monitoring Committee confirmed safety) to 30–90 mL/min/1.73 m². Initially, screening UACR between 300 and 5000 mg/g values were considered, but this was broadened to improve representation at the 1st protocol amendment (on 24 October 2023) to between 100 and 5000 mg/g. All participants were required to be treated with a renin–angiotensin system inhibitor at maximum labeled dose as tolerated for at least a month prior to screening, and were not allowed to be taking an SGLT2i or potassium lowering therapy within 8  weeks of screening. Key exclusion included serum potassium levels >4.8 mmol/L, type 1 diabetes, prior or planned kidney transplantation, chronic symptomatic heart failure with reduced ejection fraction, and a recent cardiovascular event (myocardial infarction, stroke or hospitalization for worsening heart failure) within 90 days. All participants provided written informed consent and the trial protocol was approved by all local institutional review boards or ethnic committees. The trial was overseen by an independent Data Monitoring Committee.

All study medications were continued for a treatment duration of 180 days, and then these therapies were withdrawn in blinded fashion with follow-up assessment at 210 days. There were seven scheduled study visits during which UACR, serum potassium, and serum creatinine were measured and analyzed in central laboratories. Standardized office blood pressures (after 5 min of rest) were averaged across three measurements.

### Efficacy and safety outcomes

The primary efficacy outcome was the change in UACR from baseline to 180 days. A secondary efficacy outcome was the reduction in UACR to a prespecified threshold of >30%, which is supported by the American Diabetes Association Standards of Care as a target for therapeutic optimization [12]. Adverse events potentially relevant to finerenone, empagliflozin or their combination were systematically collected and included hyperkalemia, acute kidney injury, >30% decline in eGFR at 30 ± 4 days, symptomatic hypotension, urosepsis or pyelonephritis, and genital mycotic infections.

### KDIGO risk categories

eGFR was calculated as creatinine-based on the Chronic Kidney Disease Epidemiology Collaboration equation [13] with a modification in Japanese participants [14]. First morning urine sampling of UACR was averaged over three consecutive days for screening albuminuria assessment. All individuals with available screening data for both eGFR and UACR were classified across the KDIGO risk grid. KDIGO risk categories were classified in line with clinical practice guidelines: [3] “low risk” (eGFR ≥60 mL/min/1.73 m^2^ and UACR <30 mg/g); “moderate risk” (eGFR ≥60 mL/min/1.73 m^2^ and UACR of 30–299 mg/g or eGFR 45–59 mL/min/1.73 m^2^ and UACR <30 mg/g); “high risk” (eGFR ≥60 mL/min/1.73 m^2^ and UACR ≥300 mg/g or eGFR 45–59 mL/min/1.73 m^2^ and UACR 30–299 or eGFR 30–44 mL/min/1.73 m^2^ and UACR <30 mg/g); and “very high risk” (eGFR<30 mL/min/1.73 m^2^ or eGFR <45 mL/min/1.73 m^2^ and UACR ≥30 mg/g or eGFR<60 mL/min/1.73 m^2^ and UACR ≥300 mg/g). The “low risk” and “moderate risk” categories were combined given limited sample size for analytic purposes.

### Statistical analyses

All efficacy analyses were conducted in the full analysis set which included all randomized patients (except those who were misrandomized or from sites with critical Good Clinical Practice guideline violations). All safety analyses were conducted in the safety analysis set who were validly randomized and received at least one dose of study drug.

The primary endpoint, change in UACR (which was log-transformed given skewed distribution), was analyzed using a mixed model for repeated measures and expressed as least-squares mean ratio comparing combination therapy with each monotherapy. We evaluated for potential effect modification based on baseline KDIGO risk categorization via interaction testing. Missing data were assumed in this model to be missing at random. The secondary endpoint of UACR reductions of >30% from baseline was analyzed using logistic regression models, adjusted for eGFR and UACR stratification factors, KDIGO risk categories, and its interaction with the treatment term. Only patients with baseline and 180-day values of UACR were considered for the secondary endpoint analysis. Changes from baseline in serum potassium, systolic blood pressure and eGFR were analyzed using a mixed model for repeated measures. Treatment-emergent adverse events are reported for any participant who experienced an adverse event after first dose and up to 3 days after temporary or permanent drug discontinuation.

Subgroup analyses by KDIGO risk categories were prespecified in the Academic Statistical Analysis Plan, prior to database lock. No adjustment for multiple comparisons was made. All statistical analyses were performed using SAS and *P* < .05 was considered statistically significant.

## RESULTS

**Figure 1:**
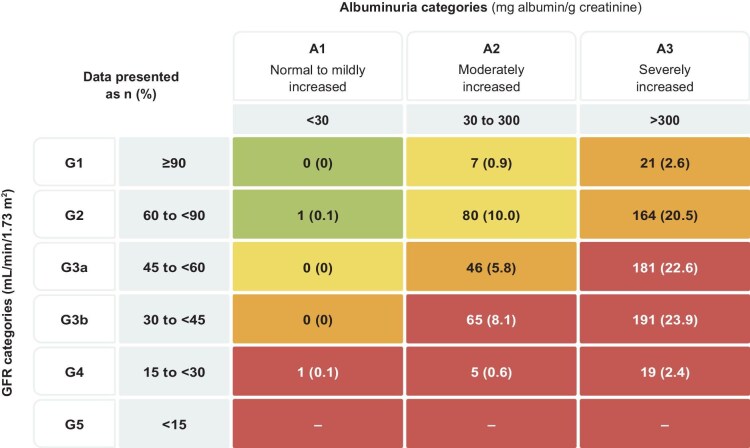
Distribution of KDIGO risk in the CONFIDENCE trial. The green and yellow coloring reflects low and moderate risk, orange coloring reflects high risk and red reflects very high risk.

From 23 June 2022 to 14 August 2024, 818 participants were randomized. Patients who were misrandomized (and did not receive a single dose of study medication) or those who were enrolled in sites where critical Good Clinical Practice guideline violations were identified were excluded from all efficacy and safety analyses (*n* = 18). Among the 781 randomized participants with available data, 88 (11.3%) were classified according to the KDIGO low/moderate risk, 231 (29.6%) to high risk, and 462 (59.2%) to very high risk categories at baseline. The KDIGO risk heat map in the CONFIDENCE trial is presented in Fig. 1. The baseline characteristics of study participants were largely comparable across KDIGO risk categories (Table 1), except KDIGO high and very high risk participants were more likely to be enrolled from Asia, and by design had lower eGFR and higher UACR levels. Across KDIGO risk categories, patients were well treated with background medical therapies including near complete use of renin–angiotensin system inhibitors, over 70% use of statins, and over 20% use of GLP-1RA.

**Table 1:** Baseline characteristics of CONFIDENCE participants by KDIGO risk category.

	Low/moderate risk (*n* = 88)	High risk (*n* = 231)	Very high risk (*n* = 462)
Age (years)	65.5 (10.4)	65.1 (10.8)	67.3 (10.0)
Men, *n* (%)	69 (78.4)	168 (72.7)	353 (76.4)
Body mass index (kg/m^2^)	30.5 (6.6)	29.6 (6.4)	28.9 (5.7)
Geographic region, *n* (%)			
Asia	22 (25.0)	116 (50.2)	220 (47.6)
Europe	28 (31.8)	62 (26.8)	120 (26.0)
North America	38 (43.2)	53 (22.9)	122 (26.4)
Baseline UACR (mg/g)^a^	191 (124, 256)	628 (327, 1186)	663 (389, 1255)
Baseline serum potassium (mmol/L)	4.41 (0.39)	4.46 (0.43)	4.51 (0.42)
Baseline eGFR (mL/min/1.73 m^2^)	73.1 (10.9)	69.5 (13.0)	43.0 (8.6)
Baseline HbA1c (%)	7.02 (1.13)	7.49 (1.23)	7.25 (1.22)
Baseline systolic blood pressure (mmHg)	133.6 (13.1)	136.8 (12.6)	134.6 (13.4)
History of atherosclerotic cardiovascular disease, *n* (%)	21 (23.9)	59 (25.5)	138 (29.9)
History of atrial fibrillation, *n* (%)	7 (8.0)	9 (3.9)	34 (7.4)
History of hypertension, *n* (%)	78 (88.6)	204 (88.3)	406 (87.9)
Statins, *n* (%)	77 (87.5)	171 (74.0)	334 (72.3)
Antiplatelets, *n* (%)	38 (43.2)	88 (38.1)	187 (40.5)
GLP-1RA, *n* (%)	20 (22.7)	59 (25.5)	97 (21.0)

Data are presented as mean (standard deviation), median (25th, 75th percentile) or *n* (%).

^a^Median (25th, 75th percentile).

HbA1c, glycated hemoglobin.

### Efficacy analyses by KDIGO risk categories

**Figure 2:**
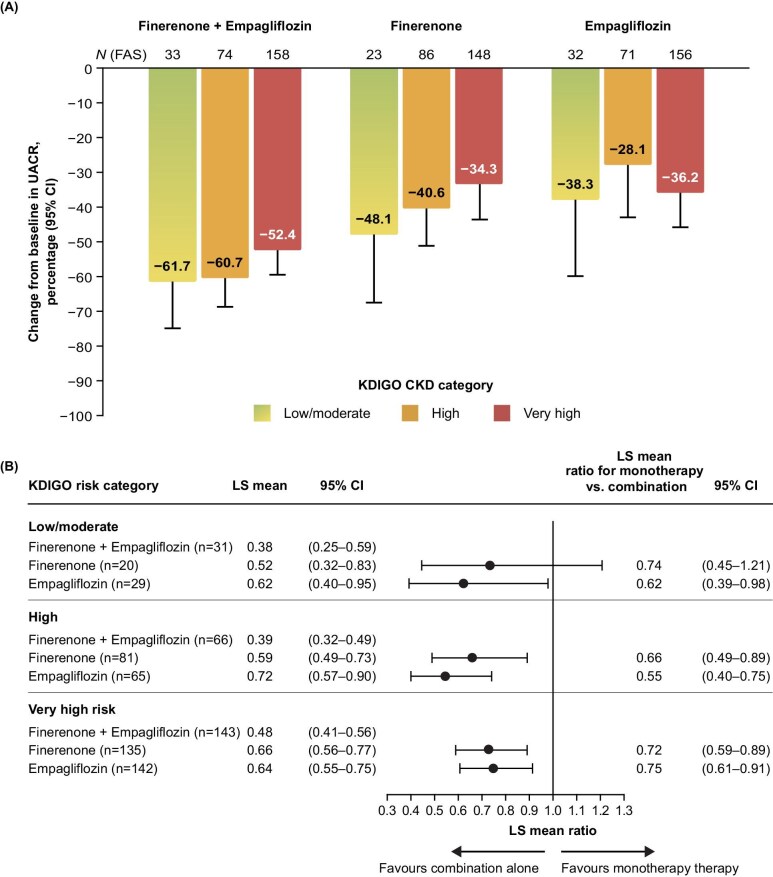
Primary efficacy endpoint analysis of change in UACR from baseline by KDIGO risk category. (**A**) Relative changes in UACR from baseline by KDIGO risk category^†,‡^. (**B**) Least squares (LS) mean ratios (with 95% CI) of change from baseline in UACR for combination therapy versus finerenone or empagliflozin alone^‡^. ^†^Percentage UACR change calculation = (LS mean ratio to baseline – 1) × 100. ^‡^Mixed model for repeated measures analysis including the following factors: treatment group, time, treatment × time, UACR category at screening, UACR baseline value and UACR baseline value × time.

Median baseline UACR values were 191 (25th–75th percentile 124, 256) mg/g in the low/moderate risk group, 628 (25th–75th percentile 327, 1186) mg/g in the high risk group, and 663 (25th–75th percentile 389, 1255) mg/g in the very high risk group. In the overall population, UACR was reduced to a greater degree with combination therapy than with either monotherapy alone, with significant effects observed as early as the first post-randomization visit at 14 days. At Day 180, combination therapy reduced UACR levels similarly across all KDIGO risk categories (−61.7% in the low/moderate-risk, −60.7% in the high-risk, and −52.4% in the very high-risk categories; Fig. 2A). Compared with finerenone alone or empagliflozin alone, combination therapy consistently resulted in greater relative reductions in UACR by Day 180 across KDIGO risk categories (*P*_interaction_ > .05; Fig. 2B). For instance, in the very high risk group (*n* = 462), the least squares mean ratio of combination therapy was 0.72 [95% confidence interval (CI) 0.59–0.89] compared with finerenone alone and 0.75 (95% CI 0.61–0.91) compared with empagliflozin alone.

**Figure 3:**
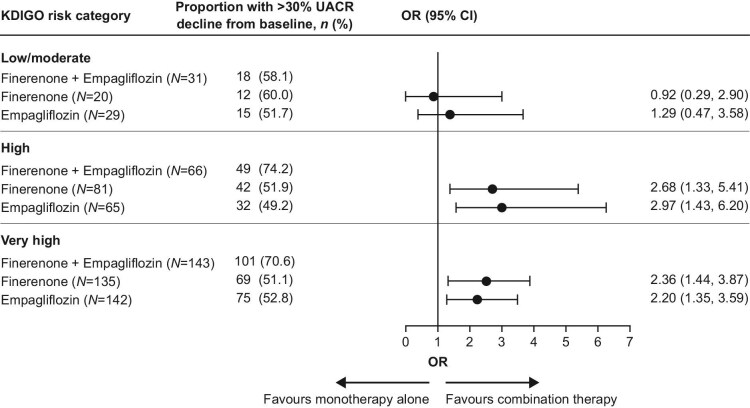
Secondary efficacy analysis of >30% reduction in UACR from baseline at Day 180, by KDIGO risk category.

Similarly, a substantial proportion of patients with simultaneous initiation of finerenone and empagliflozin experienced >30% decline in UACR from baseline to Day 180 (58.1% in the low/moderate risk, 74.2% in the high risk and 70.6% in the very high risk group). This proportion with clinically significant response was significantly greater with combination therapy compared with either monotherapy, irrespective of KDIGO risk classification (*P*_interaction_ = .27 for combination therapy vs finerenone and *P*_interaction_ = .43 for combination therapy vs empagliflozin; Fig. 3). While it appeared that the odds of achieving >30% reductions in UACR with combination therapy (compared with either monotherapy) at 180 days was somewhat attenuated in the low/moderate risk group, confidence limits were wide and no statistical significant interaction across KDIGO risk categories was observed. Furthermore, at the immediate previous visit (at 90 days), clearer benefits were seen in this subgroup further supporting instability in these estimates (proportions with >30% decline in UACR at 90 days: 66.7% with combination therapy, 43.5% with finerenone alone and 46.9% with empagliflozin alone).

### Safety analyses by KDIGO risk categories

Baseline serum potassium levels were slightly higher in the higher KDIGO risk categories (low/moderate risk: 4.41 ± 0.39 mmol/L; high risk: 4.46 ± 0.43 mmol/L; very high risk: 4.51 ± 0.42 mmol/L). Serum potassium levels rose with finerenone and the combination of finerenone and empagliflozin, with a pattern that was generally similar across KDIGO risk categories (Supplementary data, Fig. S1). Serum potassium levels returned to near-baseline in all arms across KDIGO risk categories within 30 days after drug withdrawal. The proportion of participants with any treatment-emergent hyperkalemia adverse events was generally higher with elevated KDIGO risk. The risk of treatment-emergent hyperkalemia adverse events appeared to be consistently lower with combination therapy than with finerenone monotherapy across KDIGO risk categories (Table 2).

**Table 2:** Adverse events by KDIGO risk category.

KDIGO risk category	Event	Finerenone + empagliflozin	Finerenone	Empagliflozin	Total
Low/moderate risk	≥1 adverse event	16 (48.5)	6 (26.1)	10 (31.3)	32 (36.4)
	Acute kidney injury	1 (3.0)	0	0	1 (1.1)
	Genital mycotic infection	1 (3.0)	0	2 (6.3)	3 (3.4)
	Hyperkalemia^a^	2 (6.1)	2 (8.7)	0 (0.0)	4 (4.5)
	Serum K >5.5 mmol/L^b^	4 (12.1)	3 (13.0)	1 (3.1)	8 (9.1)
	Serum K >5.5 to ≤6.0 mmol/L^b^	4 (12.1)	3 (13.0)	1 (3.1)	8 (9.1)
	Serum K >6.0 mmol/L^b^	1 (3.0)	0	0	1 (1.1)
	Urosepsis and pyelonephritis	1 (3.0)	0	0	1 (1.1)
	Symptomatic hypotension	0	0	0	0
	eGFR decline >30% at 30 days^c^	4 (12.1)	1 (4.3)	0	5 (5.7)
High risk	≥1 adverse event	25 (34.2)	35 (40.7)	14 (20.0)	74 (32.3)
	Acute kidney injury	2 (2.7)	2 (2.3)	0	4 (1.7)
	Genital mycotic infection	0	0	1 (1.4)	1 (0.4)
	Hyperkalemia^a^	7 (8.1)	5 (6.8)	2 (2.9)	14 (6.1)
	Serum K >5.5 mmol/L^b^	5 (6.8)	10 (11.6)	8 (11.4)	23 (10.0)
	Serum K >5.5 to ≤6.0 mmol/L^b^	4 (5.5)	8 (9.3)	7 (10.0)	19 (8.3)
	Serum K >6.0 mmol/L^b^	2 (2.7)	4 (4.7)	1 (1.4)	7 (3.1)
	Urosepsis and pyelonephritis	0	0	0	0
	Symptomatic hypotension	0	0	0	0
	eGFR decline >30% at 30 days^c^	6 (8.2)	5 (5.8)	1 (1.4)	12 (5.2)
Very high risk	≥1 adverse event	60 (38.0)	47 (31.8)	37 (23.7)	144 (31.2)
	Acute kidney injury	1 (0.6)	1 (0.7)	0	2 (0.4)
	Genital mycotic infection	3 (1.9)	0	1 (0.6)	4 (0.9)
	Hyperkalemia^a^	20 (13.5)	18 (11.4)	8 (5.1)	46 (10.0)
	Serum K >5.5 mmol/L^b^	31 (19.6)	33 (22.3)	16 (10.3)	80 (17.3)
	Serum K >5.5 to ≤6.0 mmol/L^b^	26 (16.5)	30 (20.3)	13 (8.3)	69 (14.9)
	Serum K >6.0 mmol/L^b^	9 (5.7)	8 (5.4)	6 (3.8)	23 (5.0)
	Symptomatic hypotension	2 (1.3)	0	0	2 (0.4)
	Urosepsis and pyelonephritis	0	0	1 (0.6)	1 (0.2)
	eGFR decline >30% at 30 days^c^	7 (4.4)	3 (2.0)	2 (1.3)	12 (2.6)

Data are presented as *n* (%).

All safety analyses were conducted in the safety analysis set who were validly randomized and received at least one dose of study drug. Treatment-emergent adverse events are reported for any participant who experienced an adverse event after first dose and up to 3 days after temporary or permanent drug discontinuation.

^a^Hyperkalemia is defined as either the adverse event preferred terms of “hyperkalemia” or “blood potassium increased.”

^b^The denominator represents all the participants at risk for an abnormal laboratory result. Participants must have had both a baseline and postbaseline (after the first dose and up to 3 days after any temporary or permanent interruption of the trial treatment) value, with the baseline value not exceeding the displayed threshold. The numerator represents the number of participants at risk who had at least one postbaseline laboratory assessment that met the criterion.

^c^The eGFR was calculated based on serum creatinine measurement at day 30 ± 4 days, and was based on the 2009 Chronic Kidney Disease Epidemiology Collaboration equation, which was modified in Japanese participants.

By design, baseline eGFR was lower in the higher KDIGO risk categories (low/moderate risk: 73.1 ± 10.9 mL/min/1.73 m^2^; high risk: 69.5 ± 13.0 mL/min/1.73 m^2^; very high risk: 43.0 ± 8.6 mL/min/1.73 m^2^). Early eGFR declines in any arm were more prominent in patients at low/moderate KDIGO risk, with minimal changes in eGFR observed in very high risk patients during follow-up (Supplementary data, Fig. S2). eGFR that declined to >30% at 30 ± 4 days after initiation of combination therapy was more common in low/moderate risk (12.1%) and high risk groups (8.2%) compared with very high risk group (4.4%). However, rates of investigator-reported acute kidney injury were infrequent (≤3%) with combination therapy across KDIGO risk categories.

Baseline systolic blood pressures were 133.6 ± 13.1 mmHg (low/moderate risk), 136.8 ± 12.6 mmHg (high risk) and 134.6 ± 13.4 mmHg (very high risk). Combination therapy resulted in an early reduction in blood pressure compared with the monotherapy arms, a pattern that was observed across KDIGO risk categories (Supplementary data, Fig. S3). Systolic blood pressure returned to near-baseline within 30 days of drug discontinuation across KDIGO risk categories. Symptomatic hypotension did not occur in any patient with low/moderate or high KDIGO risk, and was reported in two patients with very high KDIGO risk (Table 2).

## DISCUSSION

In this prespecified analysis of the CONFIDENCE trial, simultaneous initiation of the nsMRA finerenone and the SGLT2i empagliflozin consistently resulted in greater UACR lowering by 180 days than starting either monotherapy alone across KDIGO risk categories. More patients with combination therapy achieved clinically meaningful (>30%) reductions in UACR, irrespective of KDIGO risk category. Risks of hyperkalemia were generally greater at higher KDIGO risk, but combination therapy resulted in numerically lower rates of hyperkalemia compared with the finerenone arm alone in all KDIGO risk categories. eGFR declines after initiating combination therapy were much less frequent in those at very high KDIGO risk (with the lowest baseline eGFR). Blood pressure lowering was greater with combination therapy across KDIGO risk categories, but rates of symptomatic hypotension were infrequent. Taken together, these data substantiate the consistent efficacy and safety of simultaneous initiation of combination therapy with nsMRA and SGLT2i across a range of predicted risk of kidney disease progression based on KDIGO risk classification, including those at high and very high risk.

The CKD Prognosis Consortium conducted a large individual-participant data meta-analysis across 114 cohorts and demonstrated that eGFR and UACR jointly predict risk of kidney failure, a range of cardiovascular events (coronary heart disease, stroke, heart failure, atrial fibrillation and peripheral artery disease), hospitalizations, and mortality [4]. In clinical practice, the KDIGO heat map may guide decision-making regarding treatment intensification, frequency of monitoring, and the need to establish collaborative care with a nephrologist. As such, understanding the efficacy and safety of risk lowering therapies across KDIGO risk categories is of clinical importance.

The CONFIDENCE trial evaluated a broad at-risk population with type 2 diabetes and CKD. During the trial, eGFR criteria were expanded to encompass 30–90 mL/min/1.73 m² (from 40–90 mL/min/1.73 m²) after Data Monitoring Committee confirmed safety and the UACR criteria were expanded to include lower inclusion levels of UACR (down to 100 mg/g) at the first amendment. The relative UACR lowering expected with combination therapy with nsMRA and SGLT2i was consistent across KDIGO risk categories, suggesting that the forecasted risk may be similarly modified irrespective of baseline risk. More than half of participants in each of the KDIGO risk categories surpassed 30% or greater lowering in UACR, a threshold supported by guidelines for high-risk patients [12]. Indeed, the magnitude of UACR lowering is strongly correlated with subsequent risk of end-stage kidney disease, reinforcing its potential as a therapeutic target [15, 16]. Furthermore, absolute UACR lowering with combination therapy would be expected to be greater at higher KDIGO risk with many experiencing “regression” in albuminuria to UACR levels below 300 mg/g. These data suggest that many people across KDIGO risk categories may benefit from simultaneous initiation of combination therapy with nsMRA and SGLT2i. However, we acknowledge that most enrolled participants were at KDIGO high or very high risk with only a subset of individuals at lower risk. Future research and prospective investigations are required to examine whether early introduction of disease modifying therapies in KDIGO low risk individuals may prevent or delay disease progression.

Multiple therapies are now available to slow kidney disease progression in people with CKD and type 2 diabetes; however, the optimal strategy for implementation, whether simultaneous initiation or sequential step-wise initiation, is still debated. CONFIDENCE provides reassurance of the safety of simultaneous initiation of combination therapy in UACR lowering with no unexpected safety signals across KDIGO risk categories. Patients at KDIGO high and very high risk experience higher rates of hyperkalemia, in part related to slightly higher baseline potassium levels and worse kidney function, require close laboratory monitoring after initiation of combination therapy. Numerically, rates of hyperkalemia were slightly lower with combination therapy than with finerenone alone across the KDIGO risk spectrum. These observations are consistent with a previous meta-analysis of trials showing that SGLT2i consistently reduced the risk of hyperkalemia across a range of baseline kidney function [17]. Both nsMRA and SGLT2i result in an early hemodynamically mediated reduction in eGFR, and their simultaneous initiation appears to lead to an additive acute “eGFR dip.” Early eGFR changes are at least partially reversible with eGFR values approaching baseline shortly after drug discontinuation. However, these data suggest that these early eGFR changes in response to combination therapy are largely limited to those at low/moderate or high KDIGO risk, while those at very high KDIGO risk had minimal eGFR changes across study arms. These data are in keeping with prior observations from a meta-analysis of 53 randomized clinical trials enrolling 56 413 participants showing that absolute acute eGFR declines in response to renin–angiotensin system inhibitors, blood pressure lowering and SGLT2i are larger at higher baseline eGFR [18]. Patients and clinicians should be provided anticipatory guidance regarding the expected eGFR changes after therapeutic optimization, especially when baseline eGFR is higher, to limit premature drug interruption or discontinuation.

### Study limitations

Our findings should be interpreted in the context of certain limitations. CONFIDENCE had stringent inclusion and exclusion criteria that may limit the generalizability of our findings to broader, more diverse populations, especially those at low risk of kidney disease progression. While this was a prespecified secondary analysis of CONFIDENCE, the trial was not powered to evaluate treatment effects in individual subgroups based on KDIGO risk classification. Furthermore, while randomization was stratified by eGFR (<60 and ≥60 mL/min/1.73 m²) and UACR (≤850 and >850 mg/g), the dichotomization of these variables differed from KDIGO risk categories. Treatment was only continued for 180 days, so the effects of combination therapy over a longer-term horizon, including on cardiovascular and kidney outcomes, were not evaluated. Finally, KDIGO classification represents only one approach to prognostic estimation, and other more robust models and tools (such as Kidney Failure Risk Equation or CKD Prognosis Consortium) [19–21] may more optimally risk stratify this population to guide treatment decision-making.

## CONCLUSIONS

In CONFIDENCE, the relative efficacy and safety of early simultaneous combination therapy with nsMRA and SGLT2i were consistent across a broad range of predicted kidney risk.

## Supplementary Material

gfaf160_Supplemental_File

## Data Availability

Availability of the data underlying this publication will be determined according to Bayer's commitment to the EFPIA/PhRMA “Principles for responsible clinical trial data sharing.” This pertains to scope, timepoint and process of data access. As such, Bayer commits to sharing upon request from qualified scientific and medical researchers, patient-level clinical trial data, study-level clinical trial data and protocols from clinical trials in patients for medicines and indications approved in the USA and European Union (EU) as necessary for conducting legitimate research. This applies to data on new medicines and indications that have been approved by the EU and US regulatory agencies on or after 1 January 2014. Interested researchers can use www.vivli.org to request access to anonymized patient-level data and supporting documents from clinical studies to conduct further research that can help advance medical science or improve patient care. Information on the Bayer criteria for listing studies and other relevant information is provided in the member section of the portal. Data access will be granted to anonymized patient-level data, protocols and clinical study reports after approval by an independent scientific review panel. Bayer is not involved in the decisions made by the independent review panel. Bayer will take all necessary measures to ensure that patient privacy is safeguarded.
