# Supplementary material for: Simultaneous initiation of finerenone and empagliflozin across the spectrum of kidney risk in the CONFIDENCE trial
Source: Nephrol Dial Transplant. 2025 Aug 31;41(1):161–70. doi: 10.1093/ndt/gfaf160 (PMC12722168; doi:10.1093/ndt/gfaf160)
Supplement: gfaf160_Supplemental_File [file gfaf160_supplemental_file.docx]

**Supplemental Figure 1.** Change from baseline in serum potassium levels, across KDIGO risk categories


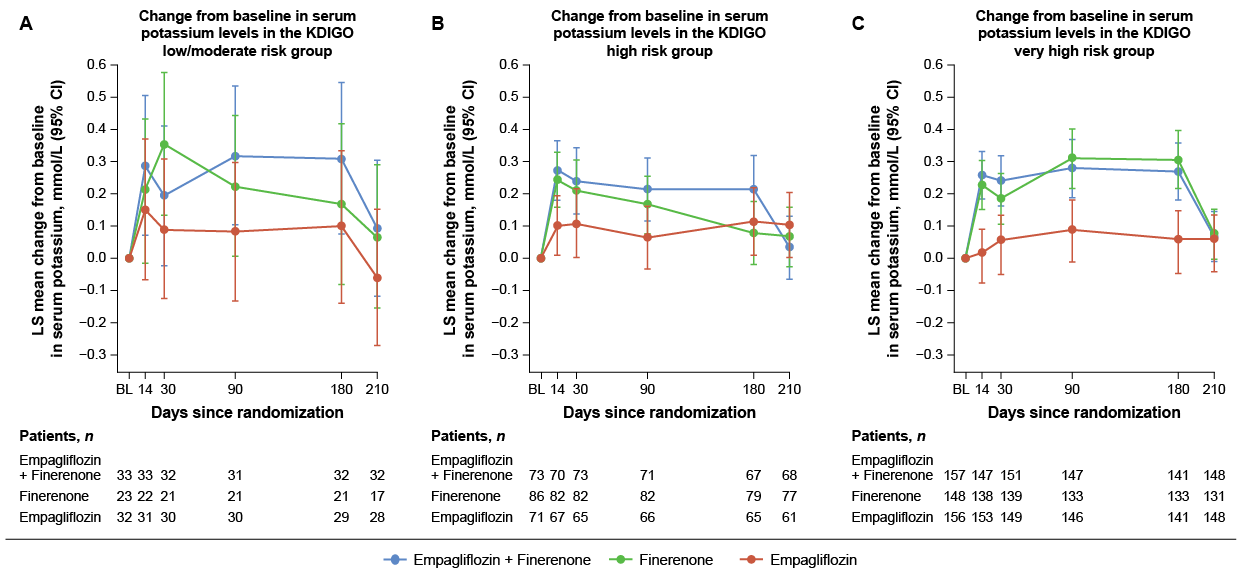


Data are presented descriptively as least squares (LS) mean changes from baseline with associated 95% confidence intervals (CI)

**Supplemental Figure 2.** Change from baseline in eGFR, across KDIGO risk categories


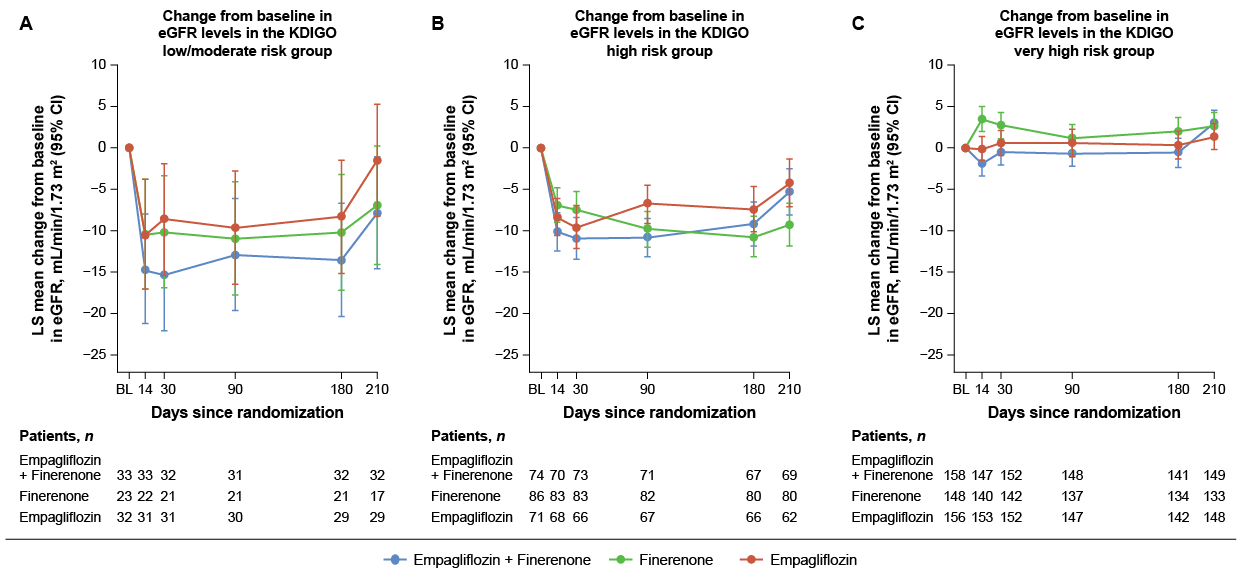


Data are presented descriptively as least squares (LS) mean changes from baseline with associated 95% confidence intervals (CI)

**Supplemental Figure 3.** Change from baseline in systolic blood pressure, across KDIGO risk categories


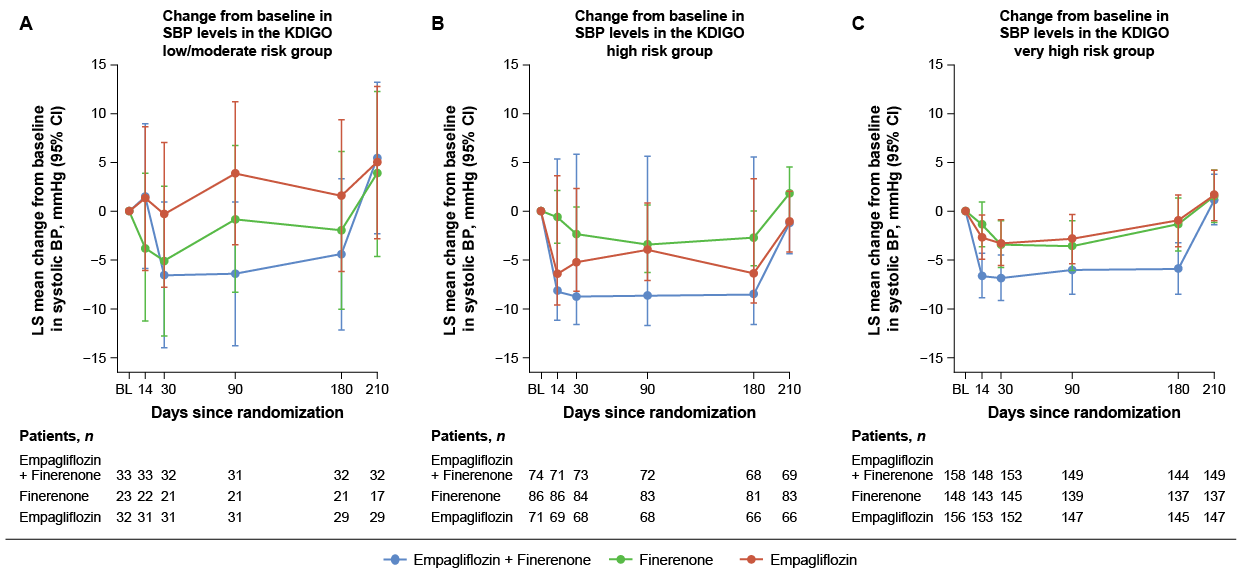


Data are presented descriptively as least squares (LS) mean changes from baseline with associated 95% confidence intervals (CI)
